# Case of Delayed Transhiatal Pancreatic Herniation After Esophagectomy

**DOI:** 10.14309/crj.0000000000000683

**Published:** 2021-11-24

**Authors:** Colin Martyn, Oluwagbenga Serrano

**Affiliations:** 1 Department of Internal Medicine, Good Samaritan Hospital, Vincennes, IN; 2 Department of Internal Medicine, Indiana University Southwest, Vincennes, IN

## CASE REPORT

Transhiatal herniation of the pancreas is a rare phenomenon, especially after esophagectomy. We present a 66-year-old White man who developed a 15-cm transhiatal hernia containing nonobstructed small bowel and pancreas approximately 23 years after an open transhiatal esophagectomy for esophageal adenocarcinoma. He experienced frequent nausea and regurgitation and slept upright in a chair for comfort for 8 years postoperatively until subsequent workup discovered an incarcerated supraumbilical incisional hernia containing omentum, which was repaired laparoscopically. Afterward, he remained asymptomatic for 15 years until he presented to our clinic with complaints of acid reflux.

**Figure 1.**
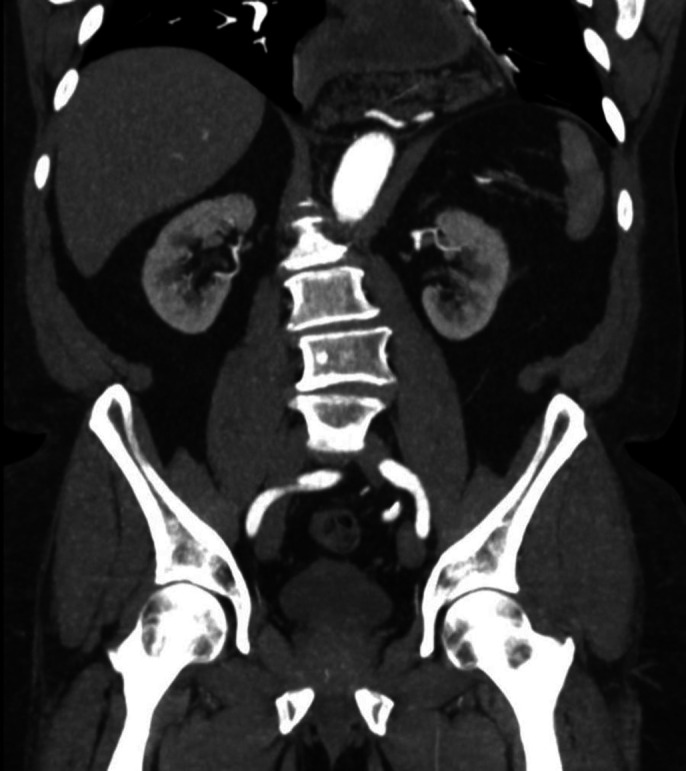
Pancreas visualized in mediastinum inferior to gastric pull-through.

**Figure 2.**
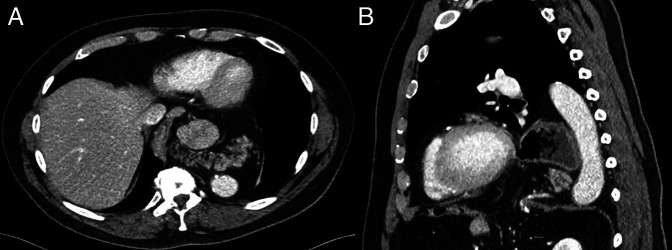
(A) Axial and (B) sagittal views of the pancreas localized posterior to gastric pull-through and anterior to the intrathoracic aorta.

Physical examination and vital signs were unremarkable aside from mild epigastric tenderness on abdominal examination. Amylase was 58 μ/L, and lipase was 30 μ/L. Thoracic, abdominal, and pelvic computed tomography demonstrated a hiatal hernia involving the gastric pull-through extending into the lower inferior mediastinum along with the pancreas and nonobstructed loops of small bowel **(**Figures 1 and 2). Our patient ultimately did not obtain a corrective surgery given the mild nature of his symptoms and the risk of disrupting the blood supply to the gastric pull-through.

Herniation of the pancreas into the mediastinum after esophagectomy is extremely rare with only 4 cases reported in the literature.^1–4^ In 1 case, the patient required emergent exploratory laparotomy secondary to bile duct obstruction.^4^ The rest were asymptomatic.^1–3^ The mechanism of pancreatic herniation into the mediastinum is believed to be secondary to stretching of the transverse mesocolon, which connects the inferior aspect of the pancreas to the transverse colon. Stretching of the transverse mesocolon during esophagectomy allows the small bowel to enter the hernia sac, and it has been suggested that it could also lengthen the posterior adhering fascia of the pancreas and allow pancreatic mediastinal herniation.^5^ Increased intra-abdominal pressure, enlargement of the hiatus, increased body mass index, and the pull of negative intrathoracic pressure all seem to increase hiatal hernia risk.

Management of postesophagectomy pancreatic herniation depends on the acuity of the hiatal hernia and symptomatology. Recommendations regarding treatment of postesophagectomy hiatal hernias involving the pancreas are largely inferred from cases of mediastinal pancreatic herniation not after esophagectomy. Some authors suggest surgical revision in all symptomatic patients without a poor prognosis because of potentially catastrophic consequences of hernia enlargement.^1,4^ However, despite hiatal hernia revision, recurrence of the hiatal hernia can be observed in up to 30% of patients.^1^

## DISCLOSURES

Author contributions: C. Martyn wrote the manuscript. O. Serrano revised the manuscript for intellectual content and is the article guarantor.

Financial disclosure: None to report.

Informed consent was obtained for this case report.
